# Reactive Disperse Dyes Bearing Various Blocked Isocyanate Groups for Digital Textile Printing Ink

**DOI:** 10.3390/molecules28093812

**Published:** 2023-04-29

**Authors:** Subin Jeong, Giyoung Kim, Hyoungeun Bae, Hyeokjin Kim, Eunjeong Seo, Sujeong Choi, Jieun Jeong, Hyocheol Jung, Sangho Lee, Inwoo Cheong, Jinchul Kim, Youngil Park

**Affiliations:** 1 Research Center for Green Fine Chemicals, Korea Research Institute of Chemical Technology, Ulsan 44412, Republic of Korea; jsubin26@krict.re.kr (S.J.); giyoung@krict.re.kr (G.K.); hebae@krict.re.kr (H.B.); ehdtka25@krict.re.kr (E.S.); crystal@krict.re.kr (S.C.); jieunj@krict.re.kr (J.J.); hjung@krict.re.kr (H.J.); slee@krict.re.kr (S.L.); 2 Test-Bed Research, Korea Dyeing and Finishing Technology Institute (DYETEC Institute), Daegu 41706, Republic of Korea; hyeokj@dyetec.or.kr; 3 Department of Applied Chemistry, Kyungpook National University (KNU), Daegu 41566, Republic of Korea

**Keywords:** digital textile printing, thermal transfer printing, reactive disperse dye, blocked isocyanate, anthraquinone dye

## Abstract

Wastewater management is of considerable economic and environmental importance for the dyeing industry. Digital textile printing (DTP), which is based on sublimation transfer and does not generate wastewater, is currently being explored as an inkjet-based method of printing colorants onto fabric. It finds wide industrial applications with most poly(ethylene terephthalate) (PET) and nylon fibers. However, for additional industrial applications, it is necessary to use natural fibers, such as cotton. Therefore, to expand the applicability of DTP, it is essential to develop a novel reactive disperse dye that can interact with the fabric. In this study, we introduced a blocked isocyanate functional group into the dye to enhance binding to the fabric. The effect of sublimation transfer on fabrics as a function of temperature was compared using the newly synthesized reactive disperse dyes with different blocking groups based on pyrazole derivatives, such as pyrazole (Py), di-methylpyrazole (DMPy), and di-*tert*-butylpyrazole (DtBPy). Fabrics coated with the new reactive disperse dyes, including PET, nylon, and cotton, were printed at 190 °C, 200 °C, and 210 °C using thermal transfer equipment. In the case of the synthesized DHP-A dye on cotton at 210 °C, the color strength was 2.1, which was higher than that of commercial dyes and other synthesized dyes, such as DMP-A and DTP-A. The fastness values of the synthesized DHP-A were measured on cotton, and it was found that the washing and light fastness values on cotton are higher than those of commercial dyes. This study confirmed the possibility of introducing isocyanate groups into reactive disperse dyes.

## 1. Introduction 

Traditional dyeing generates a large amount of wastewater, which results in environmental pollution. Hence, the focus has recently shifted to dyeing technologies that do not use water [1,2]. Among these technologies, digital inkjet printing is considered an eco-friendly and highly efficient textile printing technology that enables direct dyeing onto fabrics [3,4]. This technique reduces wastewater generation and saves energy that is otherwise needed for supplying water during screen printing. High-resolution patterning is possible using digital inkjet printing processes, the most representative of which is digital textile printing (DTP) using sublimation transfer [5,6,7].

DTP is an inkjet-based method for printing colorants onto fabrics. DTP was first reported jointly by Canon, Inc. (Tokyo, Japan) and Kanebo Textiles, Ltd. (Osaka, Japan) in 1996 and spread quickly by FESPA 2004 [8]. DTP is categorized into the direct inkjet injection method and the transfer printing method. The direct inkjet injection method exhibits high productivity by using a piezoelectric print head to directly print onto the textile [9]. Typically, it is used either for printing small designs onto garments such as T-shirts, dresses, and promotional wear, or for printing large designs onto large-format rolls of textiles, including flags, banners, and signs. Therefore, it is widely used industrially in most poly(ethylene terephthalate) (PET) and nylon fiber applications [10]. For a broader range of industrial applications, it must be applied with natural fibers, such as cotton. Existing disperse dyes exhibit limited durability in the dyeing of natural fibers. Therefore, it is necessary to develop novel reactive disperse dyes [11,12,13].

In general, reactive disperse dyes are used for printing and continuous dyeing processes, and the reactive group is selected according to the process. Reactive disperse dyes containing reactive groups such as vinyl sulfones, benzenoids, pyridines, pyrimidines, and triazines are available and utilized in various dyeing processes. For example, triazines are usually employed in direct printing, whereas the vinyl sulfone group is commonly used in two-stage and discharge printing. Monochlrorotriazine, a representative reactive and active halo aromatic group, has the disadvantage of generating acid [14]. Furthermore, the isocyanate group has the advantages of high reactivity with the hydroxyl and amine group of the functional group in the fiber and no acid generation after the reaction, and since it has never been used as a reactive group for reactive disperse dyes, it is necessary to test its capabilities in this regard.

**Scheme 1:**
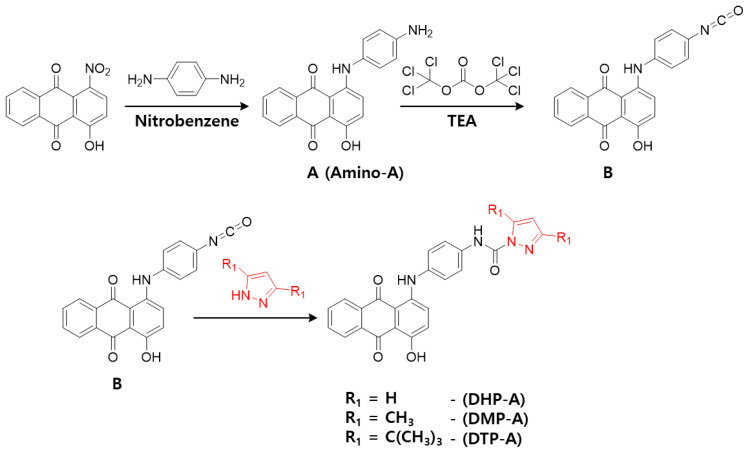
Synthetic scheme of the blocked isocyanate dyes. 1,4-Diaminobenzene and triphosgene were sequentially introduced into anthraquinone to obtain aminated compound **A** and isocyanate compound **B** in high yields. A blocking unit was attached to the resulting isocyanate derivatives **B** via an isocyanate reaction to obtain DHP-A, DMP-A, and DTP-A in high yield.

In this study, we designed and synthesized new reactive disperse dyes as digital textile printing transfer inks. The newly designed reactive disperse dyes were designed as follows (Scheme 1): (1) Using anthraquinone derivatives: Commercially available C.I. Disperse Blue 359 is a representative anthraquinone dye used as a DTP transfer ink and has excellent fastness. Therefore, new dye derivatives were synthesized based on the anthraquinone backbone; (2) Using the isocyanate group in the reactive dye: to ensure the fastness of the dyed fabric, an isocyanate group was introduced into the dyes to induce urethane bonding with the hydroxyl group of the natural fiber; (3) By the reactive blocking of isocyanate groups: Since isocyanates generally possess good reactivity, their reactivity was controlled using a pyrazole (Py) blocking group with different alkyl groups. Three types of dispersed reactive dyes with blocked isocyanate groups were synthesized, and their optical and thermal properties were measured. The synthesized dyes were applied to PET, nylon, and cotton as representative fibers through sublimation transfer. Various fastness parameters, such as the washing, rubbing, and light fastness, were measured and compared with those of commercially available sublimation transfer dyes, including C.I. Disperse Blue 359 and C.I. Disperse Blue 360.

## 2. Results

We designed and synthesized reactive disperse dyes based on anthraquinone derivatives with blocked isocyanate groups, i.e., DHP-A, DMP-A, and DTP-A, for digital textile printing. An isocyanate group in the synthesized reactive disperse dye was introduced into urethane, which bonded with the hydroxyl group of the natural fiber, such as cotton, to improve the fastness of the dyed fabric. The synthetic scheme for the new reactive disperse dyes is shown in Scheme 1. In the first step, an anthraquinone with an aromatic amine group (Amino-A, compound **A**) was synthesized with 1-hydroxy-4-nitroanthracene-9,10-dione and excess 1,4-diaminobenzene in nitrobenzene and refluxed overnight [15]. In the second step, compound B possessing an isocyanate group was synthesized using triphosgene and confirmed by a peak in the range of 2250–2280 cm^−1^ in the FT-IR spectra (Figure S1, Supplementary Materials) [16]. In the final step, the blocking agent units based on pyrazole derivatives were attached to the resulting isocyanate derivatives via an isocyanate reaction, obtaining DHP-A, DMP-A, and DTP-A in a high yield of over 80%. FT-IR spectra confirmed the disappearance of the free isocyanate peak (2250–2280 cm^−1^) of the NCO group (Figure S1). The synthesized final compounds were characterized by NMR and high-resolution mass spectrometry.

**Figure 1:**
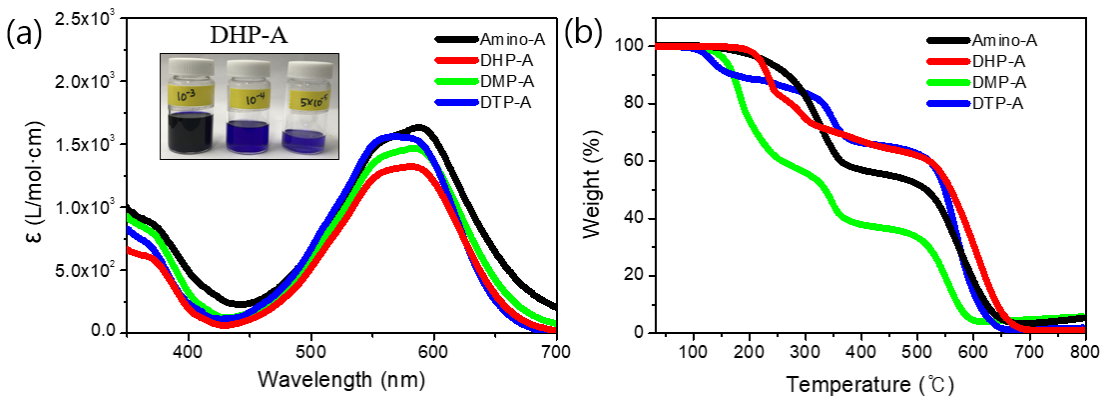
(**a**) UV–Visible absorption spectra (inset: photo of the DHP-A solution in CHCl_3_) and (**b**) TGA data of the synthesized dyes.

The optical and thermal properties of the synthesized reactive disperse dyes were characterized by UV–Vis spectroscopy and thermogravimetric analysis (TGA), the results of which are shown in Figure 1 and Table 1. In Figure 1a, the synthesized reactive disperse dyes exhibiting a blue color show the maximum absorption peaks between 570 and 588 nm and a molar absorption coefficient of more than 1.0 × 10^3^ L/mol·cm. The absorption coefficient of the reactive disperse dyes (Amino-A, DHP-A, DMP-A, and DTP-A) are 1.63, 1.32, 1.46, and 1.56 × 10^3^ L/mol·cm, respectively. When a non-chromophore, such as a pyrazole derivative, was attached to the dye, the absorption coefficient of Amino-A without a blocking group slightly decreased. In Figure 1b, TGA was used to confirm that the blocking group of the reactive disperse dye has been unblocked. The decomposition initiation temperatures of the reactive disperse dyes (DHP-A, DMP-A, and DTP-A) are 219 °C, 156 °C, and 132 °C, respectively. This means that pyrazole-blocking groups with greater steric hindrance, such as the proton, methyl and *t*-butyl groups, are significantly more effective at unblocking at lower temperatures (Figure 1b) [17,18,19].

**Figure 2:**
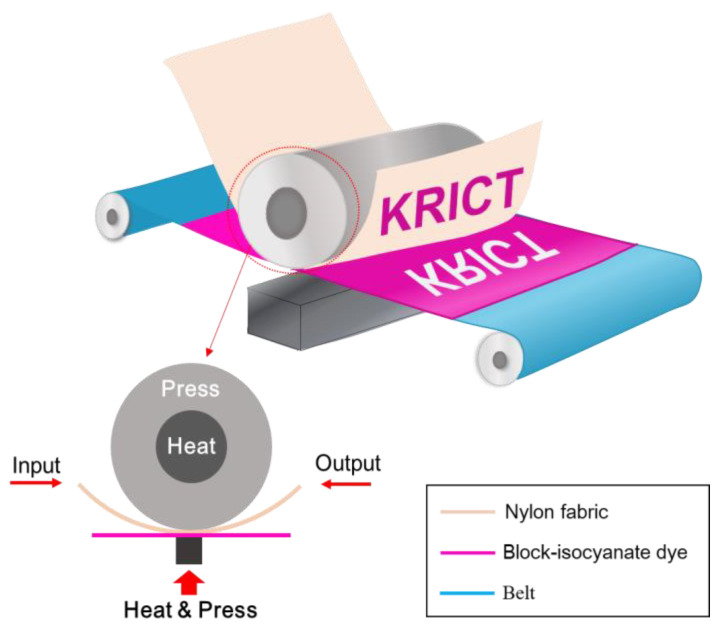
Schematic diagram of the thermal transfer printing process.

**Figure 3:**
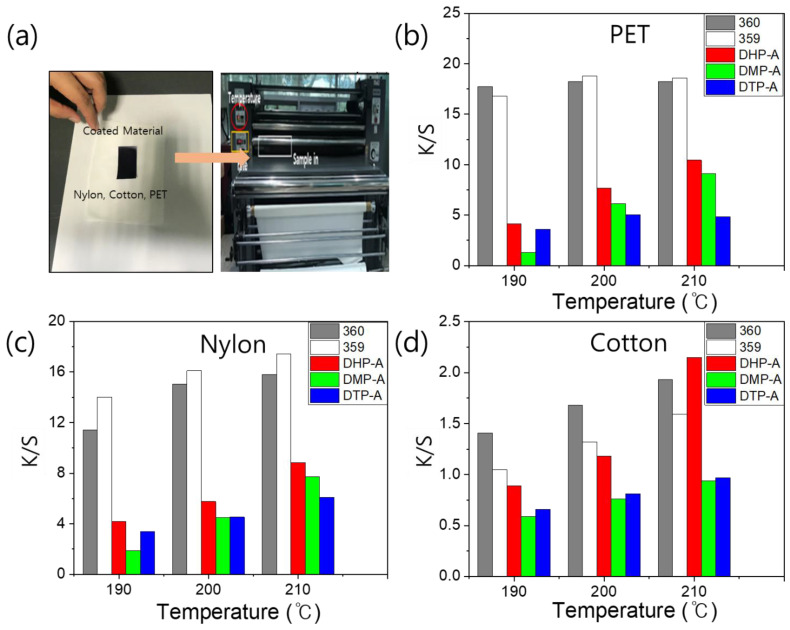
(**a**) Thermal transfer printing process; Color strength (K/S) after the thermal transfer printing process in (**b**) PET, (**c**) nylon, and (**d**) cotton at 190, 200, and 210 °C (360: C.I. Disperse Blue 360; 359: C.I. Disperse Blue 359).

Furthermore, the newly developed reactive disperse dyes underwent thermal transfer printing during the actual transfer dyeing process. The thermal transfer printing process based on roll-to-roll heat-press printing is shown in Figure 2. Both synthesized reactive disperse dyes and commercial dyes were dissolved in chloroform, and fabrics were coated using the dye-loaded solution. The coated fabrics and untreated fabrics, such as PET, nylon, and cotton, were stacked and placed in the thermal transfer equipment. Using the large drum and belt of the equipment, the fabric was printed at 190–210 °C (Figure 3a). Following thermal transfer printing on PET and nylon at 190 °C, 200 °C, and 210 °C, the results revealed that the color strength (K/S) values of the commercial dyes were generally higher (16.7–18.8 on PET and 11.4–17.4 on nylon) than those of the synthesized reactive disperse dyes (1.3–10.4 on PET and 1.9–8.8 on nylon), as shown in Figure 3a,b. However, in the case of cotton, the difference between the color strength values of the new reactive disperse dyes and commercial dyes considerably decreased with increasing temperature (Figure 3d). In particular, when the synthesized DHP-A dye was thermally transferred at 210 °C, the color strength was 2.1, which was higher than that of the commercial dyes (less than 2.0, Figure 3d) and other synthesized dyes, including DMP-A and DTP-A. These results show that DHP-A has better binding activity with cotton than all other dyes. This feature in cotton, which possesses a hydroxyl group, indicates the importance of the presence or absence of the binding site (isocyanate group) of the anthraquinone compound for binding affinity. This may be due to the pyrazole having a relatively lower boiling point (b.p. 187 °C) compared to methyl (b.p. 218 °C) and *t*-butyl pyrazole (b.p. 255 °C) derivatives, allowing the blocking group to be more efficiently removed and bound with the hydroxyl group in cotton.

**Figure 4:**
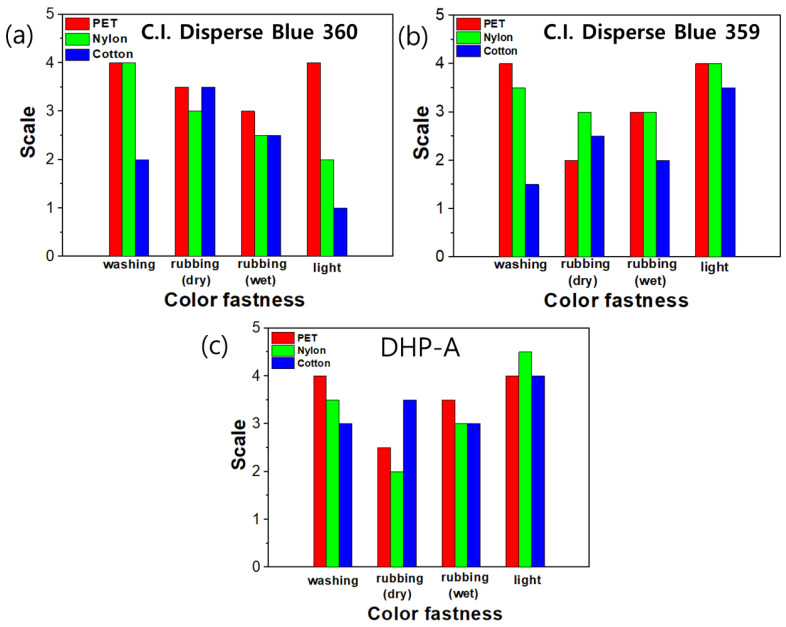
Washing, rubbing, and light fastness properties of PET, nylon, and cotton fabrics that underwent thermal transfer printing using commercial dyes: (**a**) C.I. Disperse Blue 360; (**b**) C.I. Disperse Blue 359; and the synthesized dye (**c**) DHP-A.

To measure various fastness parameters, such as the washing, rubbing, and sunlight fastness, DHP-A, which had the highest color strength among the newly synthesized dyes, was used for thermal transfer printing at 210 °C on PET, nylon, and cotton (Figure 4). The fastness values were compared with those of commercial dyes, such as C.I. Disperse Blue 359 and C.I. Disperse Blue 360. As a result of applying a commercial dye to each fiber, the PET and nylon fibers showed a relatively stable fastness of two or higher. However, in the cotton fiber, the washing fastness values of C.I. Disperse Blue 359 and C.I. Disperse Blue 360 were much lower than those of PET and nylon (Figure 4a,b). Figure 4c shows the various fastness values of the synthesized DHP-A, which has a blocked isocyanate group. Although its fastness of rubbing (dry and wet) is similar to that of commercial dyes, the other fastness parameters are superior. Notably, the washing fastness on cotton is enhanced from 1.5~2 to 3. The light fastness on cotton also increased from 1~3.5 to 4. This result means that chemical binding, such as that between the urethane of the isocyanate and the hydroxyl groups of cotton, improves the washing and light fastness.

In order to confirm that the hydroxyl group of cotton reacted with the isocyanate group of the synthesized reactive disperse dyes as described above, the synthesized DHP-A and 2-(2-(2-methoxyethoxy)ethoxy)ethan-1-ol were reacted at a thermal transfer printing temperature of 210 °C, and the results were confirmed by NMR. As shown in Figure S2, it was confirmed that pyrazole, the blocking group, was well substituted with the reactant. Therefore, this result indirectly confirmed that the isocyanate group of the synthesized dispersed reactive dyes can be well substituted with the hydroxyl group of cotton in the thermal transfer printing process.

The blocked isocyanate group of DHP-A reacted with the hydroxyl group of the reactant. Therefore, a blocked isocyanate group of the synthesized dye was introduced into the dye to improve its fastness by reacting with the hydroxyl group of cotton on digital textile printing using sublimation transfer. In conclusion, these results confirmed that a new reactive disperse dye was synthesized for the first time by introducing an isocyanate group into the reactive disperse dye and dyeing it through digital textile printing, and that transfer methods successfully improved the light and washing fastness. In this study, the introduction of an isocyanate group into these reactive disperse dyes confirmed their suitability for being used in the development of reactive disperse dyes. In the future, it is expected that it will be applicable not only to anthraquinones of reactive disperse dyes but also to other chromophores, such as azo groups.

## 3. Materials and Methods

### 3.1. Materials

1-Hydroxyl-4-nitro anthraquinone (TCI, Tokyo, Japan; >97.0%), 1,4-phenylenediamine (TCI, Tokyo; >98.0%), 4-nitrobenzene (Aldrich, St. Louis, MO, USA; >99.0%), triethylamine (Aldrich, St. Louis, MO, USA; ≥99.5%), 2,2,6,6-tetramethylheptane-3,5-dione (Aldrich, St. Louis, MO, USA; >98.0%), hydrazine hydrate monohydrate (Aldrich, St. Louis, MO, USA; >98.0%, N_2_H_4_ 64–65%), triphosgene (Aldrich, St. Louis, MO, USA; ≥98.0%), pyrazole (Aldrich, St. Louis, MO, USA; ≥98.0%), and 3,5-dimethylpyrazole (Aldrich, St. Louis, MO, USA; ≥99.0%) were purchased and used without further purification. All solvents were of ACS grade unless otherwise noted.

### 3.2. Synthesis and Characterization of Dyes

^1^H and ^13^C NMR spectra were recorded on an Ultrashield 300 MHz and 150 MHz spectrophotometer (Bruker, Germany) in CDCl_3_ and DMSO-d_6_ at room temperature. The FT-IR spectra were recorded on a Nicolet 6700/Nicolet Continuum spectrometer (Thermo Fisher Scientific, Waltham, MA, USA) in nitrogen gas conditions at room temperature. Degradation temperatures (T_d_) of compounds were measured using thermal gravimetric analysis (TGA) using a TGA4000 thermogravimetric analyzer (Perkin Elmer, Waltham, MA, USA) under the following conditions: heating rate, 20 °C/min; temperature range, 25~800 °C; nitrogen gas. The FAB^+^-mass was recorded on a JMS-700 mass spectrometer (JEOL, Tokyo, Japan).

*1-((4-Aminophenyl)amino)-4-hydroxyanthracene-9,10-dione (Amino-A)*: 1-Hydroxyl-4-nitro anthraquinone (2.00 g, 7.40 mmol) was dissolved in 60 mL of 4-nitrobenzene at 50 °C in a two-neck round-bottom flask. 1,4-Phenylenediamine (4.00 g, 36.9 mmol) was added to this solution at 150 °C. After 20 min, the reaction mixture was stirred at 210 °C for 12 h. When the mixture cooled to room temperature, ethanol was added to form a precipitate. The precipitate was filtered and washed with ethanol. The blue powder was purified using silica gel column chromatography with a mixed solvent (EtOAc/hexane, 38/62 (*v*/*v*)) as the eluent to afford a blue solid (yield 81%). ^1^H NMR (300 MHz, DMSO-d_6_): δ 13.75 (s, 1H), 11.68 (s, 1H), 8.35–8.29 (m, 2H), 8.01–7.89 (m, 2H), 7.45 (d, *J* = 9.0 Hz, 1H), 7.34 (d, *J* = 9.0 Hz, 1H), 7.03 (d, *J* = 9.0 Hz, 2H), 6.67 (d, *J* = 9.0 Hz, 2H), 5.25 (s, 2H).

3,5-*Ditertbutylpyrazole (DtBPy)*: A mixture of 2,2,6,6-tetramethylheptane-3,5-dione (1.00 g, 5.43 mmol) and hydrazine hydrate solution (0.24 g, 7.60 mmol) was poured into methanol (0.35 g, 10.8 mmol). After stirring at room temperature for 1 h, the suspension was filtered and washed several times with deionized water to afford a white solid (yield 86%). ^1^H NMR (300 MHz, CDCl3): δ (ppm) = 10.5 (br s, 1H), 5.90 (s, 1H), 1.30 (s, 18H). LC-MS: calcd. for C_11_H_20_N_2_: 180.3; found: 181.2.

*N-(4-((4-Hydroxy-9,10-dioxo-9,10-dihydroanthracen-1-yl)amino)phenyl)-1H-pyrazole-1-carboxamide (DHP-A)*: 1-((4-Aminophenyl)amino)-4-hydroxyanthracene-9,10-dione (Amino-A, 0.50 g, 1.51 mmol) and triethylamine (0.23 g, 2.27 mmol) were dissolved in 60 mL of toluene in a two-neck round-bottom flask under a N_2_ atmosphere. After a few moments, triphosgene (1.10 g, 3.70 mmol) was slowly added to the reaction solution and the reaction temperature was heated at 130 °C. The isocyanate group of the mixture was confirmed by FT-IR spectroscopy in the range of 2250–2280 cm^−1^. The desired product was obtained after the solvent was removed from the reaction mixture, proceeding to the next step without purification.

The crude reaction mixture (compound b) was cooled to 40 °C and pyrazole (0.76 g, 2 eq) was added with a small amount of toluene. The disappearance of isocyanate was confirmed of absence of the free isocyanate peak (2250–2280 cm^−1^) in the FT-IR spectra. Upon completion of the reaction, the reaction mixture was extracted with ethylacetate and water. The organic layer was dried with anhydrous MgSO_4_, filtered, and reduced, and the crude product was purified using silica gel column chromatography (EtOAc/hexane, 11/89 (*v*/*v*)) to afford a blue solid (yield 89%). ^1^H NMR (300 MHz, CDCl_3_): δ 13.74 (s, 1H), 11.81 (s, 1H), 9.15 (s, 1H), 8.40–8.34 (m, 2H), 7.84–7.77 (m, 2H), 7.70–7.57 (m, 4H), 7.32–7.20 (m, 4H), 6.49 (s, 1H); ^13^C NMR (150 MHz, CDCl_3_) δ 187.47, 157.99, 142.51, 132.79, 128.36, 126.51, 126.15, 125.86, 125.12, 125.00, 121.32, 120.98, 113.27, 77.45, 77.23, 77.03, 76.60; HRFABMS: calcd. for C_24_H_16_N_4_O_4_ [M + H]^+^ 425.1250, found 425.1246.

*N-(4-((4-Hydroxy-9,10-dioxo-9,10-dihydroanthracen-1-yl)amino)phenyl)-3,5-dimethyl-1H-pyrazole-1-carboxamide (DMP-A):* Amino-A (0.50 g, 1.51 mmol) and triethylamine (0.23 g, 2.27 mmol) were dissolved in 60 mL toluene under a N_2_ atmosphere in a two-neck round-bottom flask. After a few moments, triphosgene (1.10 g, 3.70 mmol) was slowly added to the reaction solution and the reaction temperature was heated at 130 °C. The isocyanate group in the mixture was confirmed by the presence of the peak in the range of 2250–2280 cm^−1^ in the FT-IR spectra. The desired product was obtained after the solvent was removed from the reaction mixture, proceeding to the next step without purification.

The crude reaction mixture (step b) was cooled to 40 °C and 3,5-dimethyl pyrazole (DMPy, 1.07 g, 2 eq) was added with a small amount of toluene. The disappearance of the isocyanate from the reaction mixture was confirmed by the absence of the free isocyanate peak (2250–2280 cm^−1^) in the FT-IR spectra. Upon reaction completion, the reaction mixture was extracted with ethylacetate and water. The organic layer was dried with anhydrous MgSO_4_, filtered, and reduced, and the crude product was purified using silica gel column chromatography (EtOAc/hexane, 12/88 (*v*/*v*)) to afford a blue solid (yield 88%). ^1^H NMR (300 MHz, CDCl_3_): δ 13.76 (s, 1H), 11.82 (s, 1H), 9.32 (s, 1H), 8.40–8.37 (m, 2H), 7.88–7.77 (m, 2H), 7.68–7.57 (m, 4H), 7.31–7.21 (m, 2H), 6.01 (s, 1H), 2.65 (s, 3H), 2.29 (s, 3H); ^13^C NMR (150 MHz, CDCl_3_) δ 187.46, 182.87, 158.09, 158.05, 150.55, 134.30, 133.17, 132.97, 128.34, 126.94, 126.90, 126.57, 125.92, 125.14, 125.00, 120.98, 120.96, 113.68, 110.49, 110.18, 106.14, 77.27, 77.24, 77.06, 77.03, 76.85, 76.82, 14.14, 13.99, 13.62, 11.01; HRFABMS: calcd. for C_26_H_20_N_4_O_4_ [M + H]^+^ 453.2944, found 453.2981.

*3,5-Di-tert-butyl-N-(4-((4-hydroxy-9,10-dioxo-9,10-dihydroanthracen-1-yl)amino)phenyl)-1H-pyrazole-1-carboxamide (DTP-A):* Amino-A (0.5 g, 1.5 mmol) and triethylamine (0.23 g, 2.27 mmol) were dissolved in a two-neck round-bottom flask containing 60 mL of toluene under a nitrogen atmosphere. After a few moments, triphosgene (1.10 g, 3.70 mmol) was slowly added to the reaction solution and the reaction was heated at 130 °C. The isocyanate group of the mixture was confirmed by the peak in the FT-IR spectra in the range of 2250–2280 cm^−1^. The desired product was obtained after the solvent was removed from the reaction mixture, proceeding to the next step without purification.

The crude reaction mixture (step b) was cooled to 40 °C and 3,5-di-*tert*-butyl pyrazole (DtBPy, 2.09 g, 2 eq) was added with a small amount of toluene. The absence of the free isocyanate peak (2250–2280 cm^−1^) in the FT-IR spectra confirmed that the isocyanate group was no longer present in the reaction mixture. Upon reaction completion, the reaction mixture was extracted with ethylacetate and water. The organic layer was dried with anhydrous MgSO_4_, filtered, and reduced, and the crude product was purified using silica gel column chromatography (EtOAc/hexane, 10/90 (*v*/*v*)) to afford a blue solid (yield 90%). ^1^H NMR (300 MHz, CDCl_3_): δ 13.76 (s, 1H), 11.80 (s, 1H), 9.67 (s, 1H), 8.43–8.37 (m, 2H), 7.88–7.57 (m, 6H), 7.30–7.20 (m, 2H), 6.03 (s, 1H), 1.45–1.35 (m, 18H); ^13^C NMR (150 MHz, CDCl_3_) δ 187.45, 182.81, 157.77, 156.98, 134.29, 132.94, 132.79, 128.34, 126.88, 126.51, 126.18, 126.10, 125.39, 125.22, 123.93, 121.32, 113.66, 99.20, 77.25, 77.04, 76.82, 61.43, 33.24, 32.26, 31.76, 31.60, 30.07, 29.91, 29.44, 22.67, 14.58, 14.14, 8.64; HRFABMS: calcd. for C_32_H_32_N_4_O_4_ [M + H]^+^ 537.2502, found 537.2498.

### 3.3. UV–Vis Spectrum and Color Strength

The optical absorption spectra were measured in CHCl_3_ at room temperature using a JASCO V-770 UV-VIS-NIR spectrometer in the wavelength range of 250–800 nm. The absorption efficiency was calculated using the absorption maximum wavelength of the synthesized reactive disperse dyes. The color strength (K/S) and LAB data used to determine the color difference in the black color transfer printing of nylon were measured with a color measurement instrument Ci7600 Spectrophotometer (X-rite Co. Ltd., Grand Rapids, MI, USA). The surface reflectance was measured at an interval of 10 nm in the range of 360–750 nm, which corresponds to the visible–light wavelength region. The measured surface reflectance was expressed as a K/S value based on the Kubelka–Munk equation, which describes the relationship between light absorption and scattering [20,21,22]. Measurements were performed using a 10° standard observer under D65 standard light illumination.

(1) KS=(1−Rλ)22Rλ

Here K is the absorption coefficient of the dyed fabric, which is proportional to the dye concentration; S is the diffusion coefficient of the dyed fabric, a constant determined by the dyed object; and R_λ_ is the surface reflectance of the dyed fabric to light of wavelength λ. The ratio of the absorption coefficient to the scattering coefficient indicated that it was inversely proportional to the surface reflectance of the dyed fabric.

### 3.4. Thermal Transfer Printing

Thermal transfer printing proceeded in the following order: fabric treatment, printing on transfer paper, and roll-to-roll heat-press printing. For the treatment, four types of 10% concentration surface treatment agents were prepared, and the nylon fabric was immersed in the agents by padding the coating to perform surface treatment. Padding treatment was performed at 1.2 m/min and 2 bar using Padding Mangle, DL-2005 V equipment (Daelim Starlet, Republic of Korea). Using Tenter laboratory equipment (Mathis, Switzerland), it was dried for 180 s at 180 °C to obtain nylon fabric treated with an acrylic polymer surface. To proceed with printing on transfer paper, 100% black inks (JS-100, InkTec, Republic of Korea) were printed in four passes at 360 × 720 dpi resolution on transfer paper (75 g/m^2^, Puleungyoyeog, Republic of Korea) using dispersed ink printer equipment FJ-740 K (Roland, Japan). Blue ink is a solution in which water, dispersed dyes, and a dispersant with black color are mixed.

Finally, four types of surface-treated and untreated nylon fabrics were stacked with transfer paper printed with dispersed black ink and placed in thermal transfer equipment (Sport Race, Myung-shin Engineering, Republic of Korea). Using the large drum and belt of the equipment, the nylon fabric was printed under a high temperature (195 °C) and pressure (0.3 MPa).

### 3.5. Fastness of Dyeing Fiber (Washing, Rubbing, and Light Fastness)

The fastness parameters of the blue-printed PET, nylon, and cotton samples were analyzed in terms of washing, rubbing, and light fastness. Each fastness test method was approved and the results were confirmed through an accredited certification authority. The washing fastness was evaluated based on KS K ISO 105-C06:2010, the rubbing fastness was evaluated based on KS K ISO 105-X12:2016, and the light fastness was evaluated based on KS K ISO 105-B02:2014. The washing fastness test was performed with six multi-cloths (acetate, cotton, nylon, polyester, acrylic, and wool) at 40 °C for 30 min using 0.4% ECE standard detergent, 1 g of sodium perborate, and 10 beads. The degree of contamination was determined by observing the dyed nylon and its color change. The rubbing fastness test was performed based on the degree of contamination. The printed nylon was scrubbed 10 times in 10 s with dried and wet white cloths of 1.5 mm diameter to a distance of 100 mm with a 900 g load. The light fastness was determined by the color change after exposing the printed nylon to a Xe arc lamp for 20 h. The results were graded between 1 and 5, with grade 5 being the highest value [22,23,24,25].

## 4. Conclusions

In this study, novel reactive disperse dyes (DHP-A, DMP-A, and DTP-A) were synthesized based on an anthraquinone backbone with blocked isocyanate-group pyrazole derivatives, such as pyrazole, di-methylpyrazole, and di-*tert*-butylpyrazole, respectively, as the DTP transfer ink. The new reactive disperse dyes are blue dyes with ε_max_ values of more than 1.3 × 10^3^ L·mol^−1^·cm^−1^. The effect of sublimation transfers on fabrics was investigated at 190, 200, and 210 °C using the newly synthesized reactive disperse dyes. In particular, in the case of cotton, the color strength of DHP-A dye was thermally transferred at a temperature of 210 °C higher compared to that of the commercial dyes (C.I. Disperse Blue 359 and C.I. Disperse Blue 360) and other synthesized dyes, including DMP-A and DTP-A. To measure various fastness parameters, such as the washing, rubbing, and sunlight fastness on the PET, nylon, and cotton fibers, DHP-A exhibited superior washing and light fastness values compared to those of commercial dyes. Importantly, we confirmed that the isocyanate group of DHP-A reacts with the hydroxyl group of cotton, thus increasing the washing and light fastness. Furthermore, it is important that new reactive disperse dyes can be developed and applied to various dyeing fields in the future by introducing isocyanate groups into various dye structures.

## Figures and Tables

**Table 1:** Optical properties and thermal properties of the synthesized dyes.

Synthetic Dyes	Solution in CHCl_3_		Thermal Properties
	UV_max_ (nm)	$\varepsilon_{\max}$ (L/mol·cm)	T_d_ at 5% Loss (^o^C)
Amino-A	588	1.63 × 10^3^	228
DHP-A	582	1.32 × 10^3^	219
DMP-A	585	1.46 × 10^3^	156
DTP-A	570	1.56 × 10^3^	132

## Data Availability

Not applicable.

## Supplementary Materials

The following supporting information can be downloaded at: https://www.mdpi.com/1420-3049/28/9/3812/s1, Figure S1: Scheme and FT-IR spectra of isocyanate reaction and confirmed the disappearance of the free isocyanate peak (2250–2280 cm^−1^) of NCO group.; Figure S2: ^1^H NMR spectrum of the mixture (after wasing with ethanol and dry) of the reaction of DHP-A with 2-(2-(2-methoxyethoxy)ethoxy)ethan-1-ol at 210 °C for 10 min.; Figure S3: H-NMR spectrum of DHP-A.; Figure S4: H-NMR spectrum of DMP-A.; Figure S5: H-NMR spectrum of DTP-A.

## Sample Availability

Samples of the compounds are available from the authors.

## References

[1] Kumar P., Prasad B., Mishra I., Chand S. (2008). Treatment of composite wastewater of a cotton textile mill by thermolysis and coagulation. J. Hazard. Mater..

[2] Baeta B., Ramos R.L., Lima D.R.S., Aquino S. (2012). Use of submerged anaerobic membrane bioreactor (SAMBR) containing powdered activated carbon (PAC) for the treatment of textile effluents. Water Sci. Technol..

[3] Han M., Kwon W., Park S., Jeong E. (2020). Optimization of self-crosslinking comonomer composition of polymer binder for DTP pigment ink. Text. Color. Finish..

[4] Thompson K.L. (2016). Digital Textile Printing: Colorfastness of Reactive Inks versus Pigment Inks. Master’s Thesis.

[5] Leelajariyakul S., Noguchi H., Kiatkamjornwong S. (2008). Surface-modified and micro-encapsulated pigmented inks for ink jet printing on textile fabrics. Prog. Org. Coat..

[6] Ren J., Chen G., Li X. (2017). A fine grained digital textile printing system based on image registration. Comput. Ind..

[7] Elgammal M., Schneider R., Gradzielski M. (2016). Development of self-curable hybrid pigment inks by miniemulsion polymerization for inkjet printing of cotton fabrics. Dyes Pigment..

[8] Choi S., Cho K.H., Namgoong J.W., Kim J.Y., Yoo E.S., Lee W., Jung J.W., Choi J. (2019). The synthesis and characterization of the perylene acid dye inks for digital textile printing. Dyes Pigm..

[9] Javoršek D., Javoršek A. (2011). Colour management in digital textile printing. Color. Technol..

[10] Al-Etaibi A.M., El-Apasery M.A. (2019). Dyeing performance of disperse dye on polyester fabrics using eco-friendly car-rier and their antioxidant and anticancer activities. Int. J. Environ. Res. Public Health.

[11] Sadeghi-Kiakhani M., Safapour S. (2016). Improvement of dyeing and antimicrobial properties of nylon fabrics modified using chitosan-poly(propylene imine) dendreimer hybrid. J. Ind. Eng. Chem..

[12] Tang A.Y.L., Lee C.H., Wang Y.M., Kan C.W. (2019). Dye-ing-cotton-with-reactive-dyesa-comparison-between-conventional-water-based-and-solvent-assisted-PEG-basedreverse-micellar-dyeing-systems. Cellulose.

[13] Choi G.-M., Kim K.-H. (2018). A study of the color reproducibility and color fastness of digital textile printing for nylon sublimation transfer. Res. J. Costume Cult..

[14] Taylor J.A. (2000). Recent developments in reactive dyes. Color. Technol..

[15] Shan B., Tong X., Xiong W., Qiu W., Tang B., Lu R., Ma W., Luo Y., Zhang S. (2015). A new kind of H-acid mono-azo-anthraquinone reactive dyes with surprising colour. Dyes Pigm..

[16] Satake A., Ishizawa Y., Katagiri H., Kondo S.I. (2016). Chloride Selective Macrocyclic Bisurea Derivatives with 2,2′-Binaphthalene Moieties as Spacers. J. Org. Chem..

[17] Rolph M.S., Markowska A.L.J., Warriner C.N., O’Reilly R.K. (2016). Blocked Isocyanates: From Analytical and Experimental Considerations to Non-polyurethane Applications. Polym. Chem..

[18] Wicks D.A., Wicks Z.W. (1999). Blocked isocyanates III: Part A. Mechanisms and chemistry. Prog. Org. Coat..

[19] Mühlebach A. (1994). Pyrazoles—A novel class of blocking agents for isocyanates. J. Polym. Sci. Part A Polym. Chem..

[20] Yang R., Xu Y., Xie C., Wang H. (2019). Kubelka-Munk double constant theory of digital rotor spun color blended yarn. Dyes Pigment..

[21] Xie K., Tang J., Fu D., Zhang H., Gao A. (2018). Preparation of color polymeric nanomaterials containing dispersed dye by sunflower oil synergy and color transfer property by digital inkjet printing. Prog. Org. Coat..

[22] Ghanavatkar C.W., Mishra V.R., Sekar N. (2020). Benzothiazole-pyridone and benzothiazole-pyrazole clubbed emissive azo dyes and dyeing application on polyester fabric UPF, biological, photophysical and fastness properties with correlative computational assessments. Spectrochim. Acta A. Mol. Biomol. Spectrosc..

[23] Vodyanitskii Y.N., Savichev A.T. (2017). The influence of organic matter on soil color using the regression equations of optical parameters in the system CIE- L*a*b*. Ann. Agrar. Sci..

[24] Razvan G., Pérez M.M., Herrera L.J., Rivas M.J., Yebra A., Paravina R.D. (2010). Color difference thresholds in dental ceramics. J. Dent..

[25] Pawar A.B., More S.P., Adivarekar R.V. (2017). Dyeing of Polyester and Nylon with Semi-synthetic Azo Dye by Chemical Modification of Natural Source Areca Nut. Nat. Prod. Bioprospec..

