# Supplementary material for: Reactive Disperse Dyes Bearing Various Blocked Isocyanate Groups for Digital Textile Printing Ink
Source: Molecules. 2023 Apr 29;28(9):3812. doi: 10.3390/molecules28093812 (PMC10179864; doi:10.3390/molecules28093812)
Supplement: Supplementary file 1 [file molecules-28-03812-s001.zip › molecules-2299577-supplementary.pdf]

Supplementary Information

Reactive Disperse Dyes Bearing Various Blocked Isocyanate Groups for Digital Textile Printing Ink

Subin Jeong [1,†], Gi Young Kim [1,†], Hyoung Eun Bae [1,†], Hyeok-Jin Kim [2], Eun Jeong Seo [1], Su Jeong Choi [1],
Ji-Eun Jeong [1], Hyocheol Jung [1], Sang-Ho Lee [1], In Woo Cheong [3,*], Jin Chul Kim [1,*] and Young Il Park [3,*]

[1] Research Center for Green Fine Chemicals, Korea Research Institute of Chemical Technology, Ulsan 44412, Republic of Korea
[2] Test-bed research, Korea Dyeing and Finishing Technology Institute (DYETEC Institute), Daegu 41706, Republic of Korea
[3] Department of Applied Chemistry, Kyungpook National University (KNU), Daegu 41566, Republic of Korea
* Correspondence: inwoo@knu.ac.kr (I.W.C.); jckim81@krict.re.kr (J.C.K.); ypark@krict.re.kr (Y.I.P.)
† These authors contributed equally to this work.

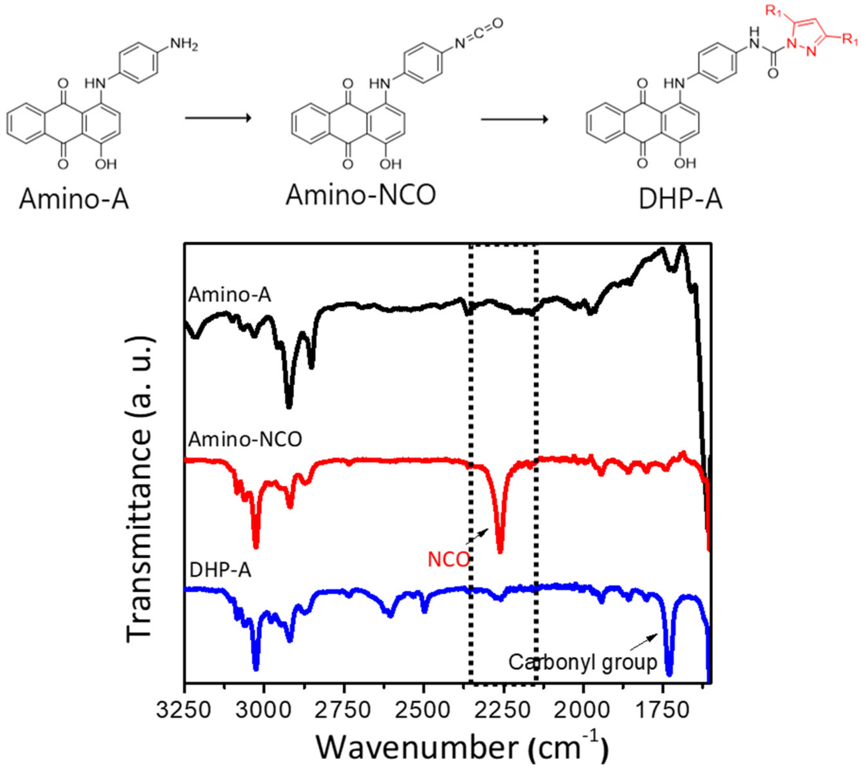

Figure S1. Scheme and FT-IR spectra of isocyanate reaction and confirmed the disappearance of the free isocyanate peak (2250-2280 cm^{-1}) of NCO group.

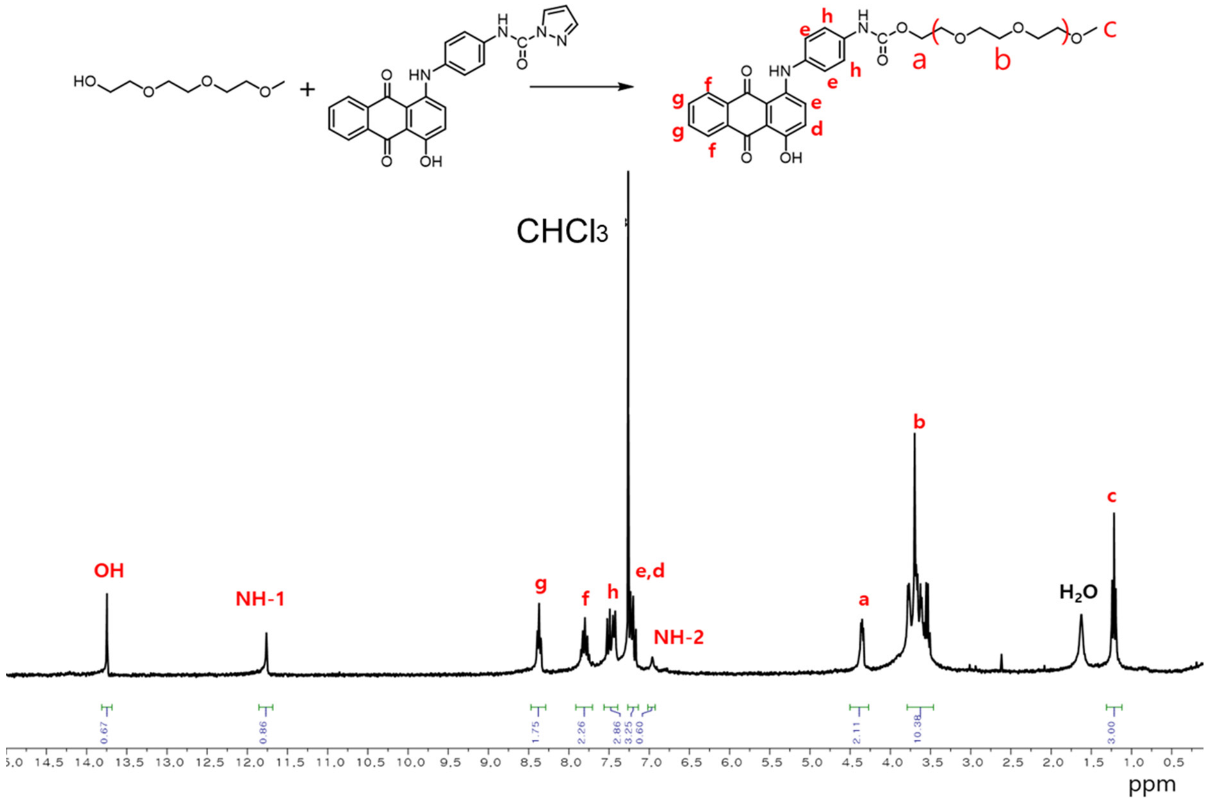

Figure S2. ^{1}H NMR spectrum of the mixture (after wasing with ethanol and dry) of the reaction of DHP-A with 2-(2-(2-methoxyethoxy)ethoxy)ethan-1-ol at 210 ◦C for 10 min.

Synthetic scheme of blocked isocyanate dyes

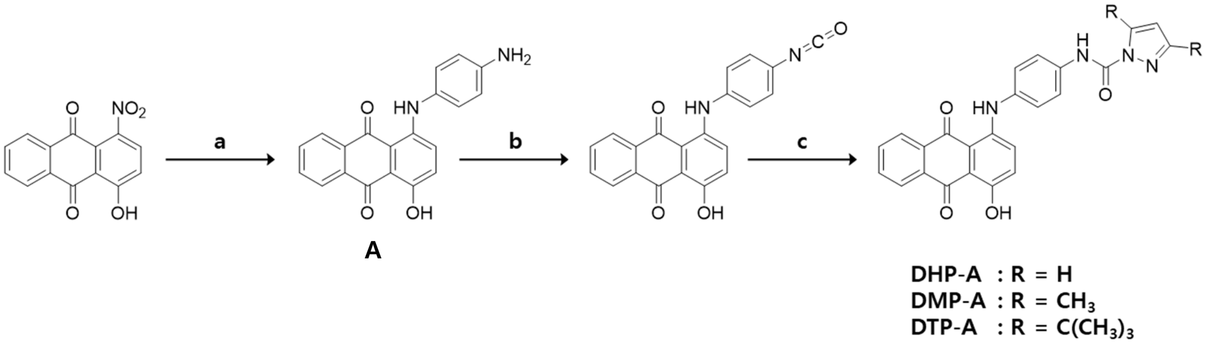

a) 1,4-Diaminobenzene, Nitrobenezene, 150 °C; b) Triethylamine, Triphosgene, 130 °C; c) Pyrazole derivated compounds (pyrazole, dimethyl pyrazole, di-*tert*-butyl pyrazole), toluene 40 °C.

1-((4-Aminophenyl)amino)-4-hydroxyanthracene-9,10-dione (Amino-A, Compound A): 1-Hydroxyl-4-nitro anthraquinone (2.00 g, 7.40 mmol) was dissolved in 60 mL of 4-nitrobenzene at 50 °C in a two-neck round-bottom flask. 1,4-Phenylenediamine (4.00 g, 36.9 mmol) was added to this solution at 150 °C. After 20 min, the reaction mixture was stirred at 210 °C for 12 h. When the mixture cooled to room temperature, ethanol was added to form a precipitate. The precipitate was filtered and washed with ethanol. The blue powder was purified using silica gel column chromatography with a mixed solvent (EtOAc/hexane, 38/62 (v/v)) as the eluent to afford a blue solid (yield 81%). **^{1}H NMR** (300 MHz, DMSO-d_6): δ 13.75 (s, 1H), 11.68 (s, 1H), 8.35-8.29 (m, 2H), 8.01-7.89 (m, 2H), 7.45 (d, J = 9.0 Hz, 1H), 7.34 (d, J = 9.0 Hz, 1H), 7.03 (d, J = 9.0 Hz, 2H), 6.67 (d, J = 9.0 Hz, 2H), 5.25 (s, 2H).

N-(4-((4-Hydroxy-9,10-dioxo-9,10-dihydroanthracen-1-yl)amino)phenyl)-1H-pyrazole-1-carboxamide (DHP-A): 1-((4-Aminophenyl)amino)-4-hydroxyanthracene-9,10-dione (Amino-A, 0.50 g, 1.51 mmol) and triethylamine (0.23 g, 2.27 mmol) were dissolved in 60 mL of toluene in a two-neck round-bottom flask under a N_2 atmosphere. After a few moments, triphosgene (1.10 g, 3.70 mmol) was slowly added to the reaction solution and the reaction temperature was heated at 130 °C. The isocyanate group of the mixture was confirmed by FT-IR spectroscopy in the range of 2,250–2,280 cm^{-1}. The desired product was obtained after the solvent was removed from the reaction mixture, proceeding to the next step without purification.

The crude reaction mixture was cooled to 40 °C and pyrazole (0.76 g, 2 eq) was added with a small amount of toluene. The disappearance of isocyanate was confirmed of absence of the free isocyanate peak (2,250–2,280 cm^{-1}) in the FT-IR spectra. Upon completion of the reaction, the reaction mixture was extracted with ethylacetate and water. The organic layer was dried with anhydrous $MgSO_4$, filtered, and reduced, and the crude product was purified using silica gel column chromatography (EtOAc/hexane, 11/89 (v/v)) to afford a blue solid (yield 89%). **^{1}H NMR** (300 MHz, $CDCl_3$): δ 13.74 (s, 1H), 11.81 (s, 1H), 9.15 (s, 1H), 8.40-8.34 (m, 2H), 7.84-7.77 (m, 2H), 7.70-7.57 (m, 4H), 7.32-7.20 (m, 4H), 6.49 (s, 1H); **^{13}C NMR** (150 MHz, $CDCl_3$) δ 187.47, 157.99, 142.51, 132.79, 128.36, 126.51, 126.15, 125.86, 125.12, 125.00, 121.32, 120.98, 113.27, 77.45, 77.23, 77.03, 76.60; HRFABMS: calcd. for $C_{24}H_{16}N_4O_4$ $[M+H]^+$ 425.1250, found 425.1246.

N-(4-((4-Hydroxy-9,10-dioxo-9,10-dihydroanthracen-1-yl)amino)phenyl)-3,5-dimethyl-1H-pyrazole-1-carboxamide (DMP-A): Amino-A (0.50 g, 1.51 mmol) and triehtylamine (0.23 g, 2.27 mmol) were dissolved in 60 mL toluene under a N_2 atmosphere in a two-neck round-bottom flask. After a few moments, triphosgene (1.10 g, 3.70 mmol) was slowly added to the reaction solution and the reaction temperature was heated at 130 °C. The isocyanate group in the mixture was confirmed by the presence of the peak in the range of 2,250–2,280 cm^{-1} in the FT-IR spectra. The desired product was obtained after the solvent was removed from the reaction mixture, proceeding to the next step without purification.

The crude reaction mixture (step b) was cooled to 40 °C and 3,5-dimethyl pyrazole (DMPy, 1.07 g, 2 eq) was added with a small amount of toluene. The disappearance of the isocyanate from the reaction mixture was confirmed by the absence of the free isocyanate peak (2,250–2,280 cm^{-1}) in the FT-IR spectra. Upon reaction completion, the reaction mixture was extracted with ethylacetate and water. The organic layer was dried with anhydrous $MgSO_4$, filtered, and reduced, and the crude product was purified using silica gel column chromatography (EtOAc/hexane, 12/88 (v/v)) to afford a blue solid (yield 88%). **^{1}H NMR** (300 MHz, $CDCl_3$): δ 13.76 (s, 1H), 11.82 (s, 1H), 9.32 (s, 1H), 8.40-8.37 (m, 2H), 7.88-7.77 (m, 2H), 7.68-7.57 (m, 4H), 7.31-7.21 (m, 2H), 6.01 (s, 1H), 2.65 (s, 3H), 2.29 (s, 3H); **^{13}C NMR** (150 MHz, $CDCl_3$) δ 187.46, 182.87, 158.09, 158.05, 150.55, 134.30, 133.17, 132.97, 128.34, 126.94, 126.90, 126.57, 125.92, 125.14, 125.00, 120.98, 120.96, 113.68, 110.49, 110.18, 106.14, 77.27, 77.24, 77.06, 77.03, 76.85, 76.82, 14.14, 13.99, 13.62, 11.01; HRFABMS: calcd. for $C_{26}H_{20}N_4O_4$ $[M+H]^+$ 453.2944, found 453.2981

3,5-Di-tert-butyl-N-(4-((4-hydroxy-9,10-dioxo-9,10-dihydroanthracen-1-yl)amino)phenyl)-1H-pyrazole-1-carboxamide (DTP-A): Amino-A (0.5 g, 1.5 mmol) and triethylamine (0.23 g, 2.27 mmol) were dissolved in a two-neck round-bottom flask containing 60 mL of toluene under a nitrogen atmosphere. After a few moments, triphosgene (1.10 g, 3.70 mmol) was slowly added to the reaction solution and the reaction was heated at 130 °C. The isocyanate group of the mixture was confirmed by the peak in the FT-IR spectra in the range of 2,250–2,280 cm^{-1}. The desired product was obtained after the solvent was removed from the reaction mixture, proceeding to the next step without purification.

The crude reaction mixture (step b) was cooled to 40 °C and 3,5-di-*tert*-butyl pyrazole (DtBPy, 2.09 g, 2 eq) was added with a small amount of toluene. The absence of the free isocyanate peak (2,250–2,280 cm^{-1}) in the FT-IR spectra confirmed that the isocyanate group was no longer present in the reaction mixture. Upon reaction completion, the reaction mixture was extracted with ethylacetate and water. The organic layer was dried with anhydrous $MgSO_4$, filtered, and reduced, and the crude product was purified using silica gel column chromatography (EtOAc/hexane, 10/90 (v/v)) to afford a blue solid (yield 90%). **^{1}H NMR** (300 MHz, $CDCl_3$): δ 13.76 (s, 1H), 11.80 (s, 1H), 9.67 (s, 1H), 8.43-8.37 (m, 2H), 7.88-7.57 (m, 6H), 7.30-7.20 (m, 2H), 6.03 (s, 1H), 1.45-1.35 (m, 18H); **^{13}C NMR** (150 MHz, $CDCl_3$) δ 187.45, 182.81, 157.77, 156.98, 134.29, 132.94, 132.79, 128.34, 126.88, 126.51, 126.18, 126.10, 125.39, 125.22, 123.93, 121.32, 113.66, 99.20, 77.25, 77.04, 76.82, 61.43, 33.24, 32.26, 31.76, 31.60, 30.07, 29.91, 29.44, 22.67, 14.58, 14.14, 8.64; HRFABMS: calcd. for $C_{32}H_{32}N_4O_4$ $[M+H]^+$ 537.2502, found 537.2498

NMR spectrum of blocked isocyanate dyes

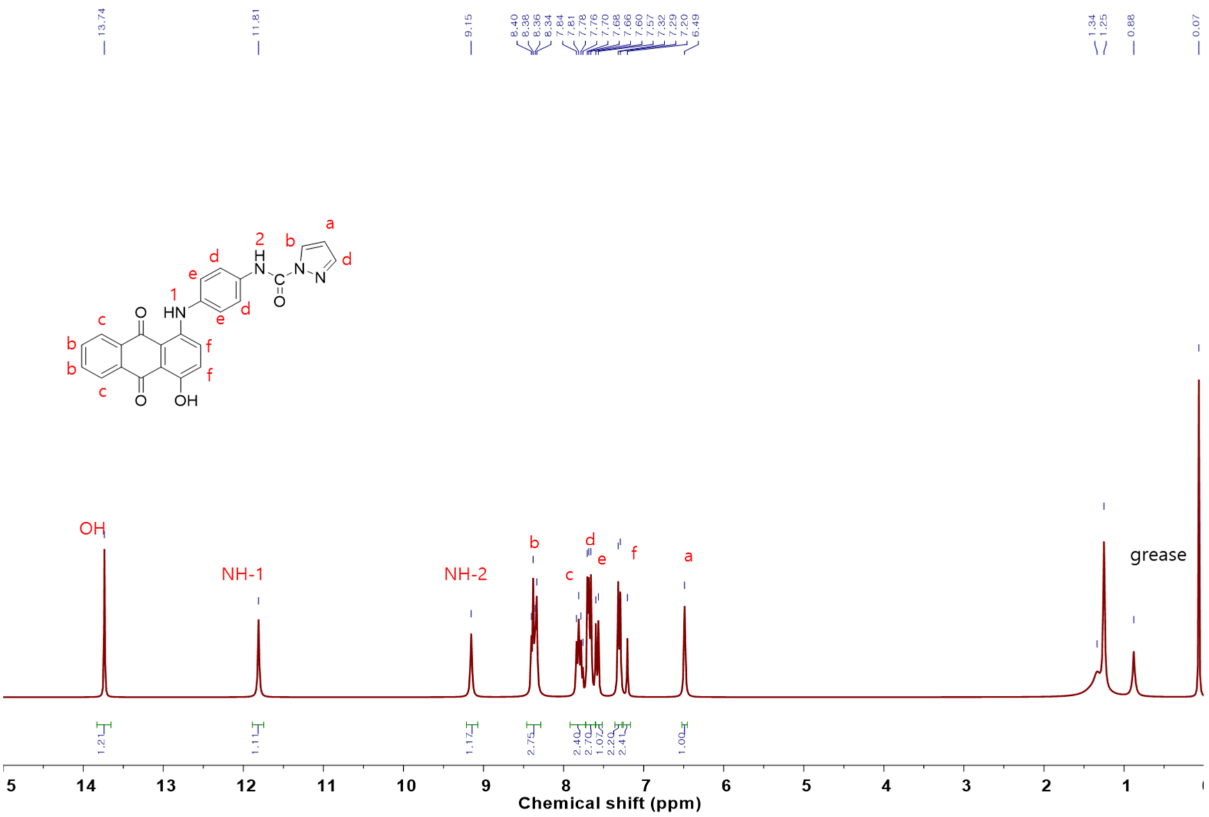

Figure S3. H-NMR spectrum of DHP-A

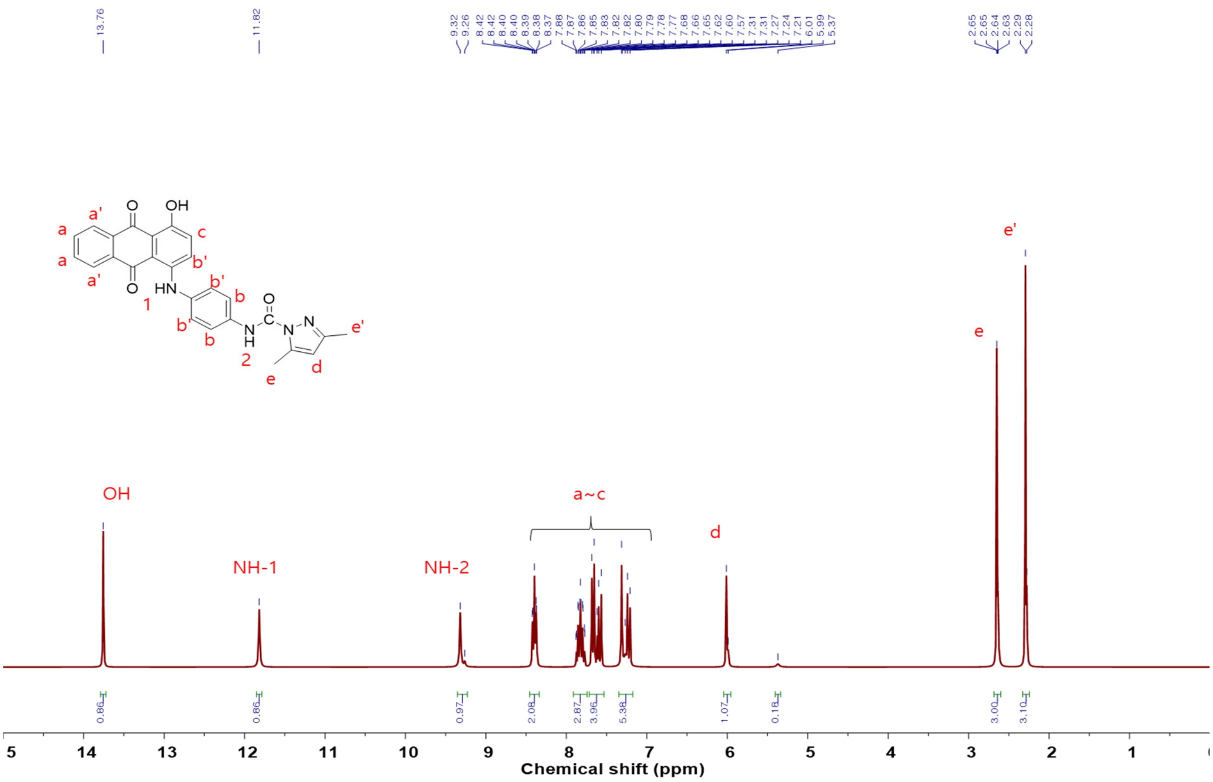

Figure S4. H-NMR spectrum of DMP-A

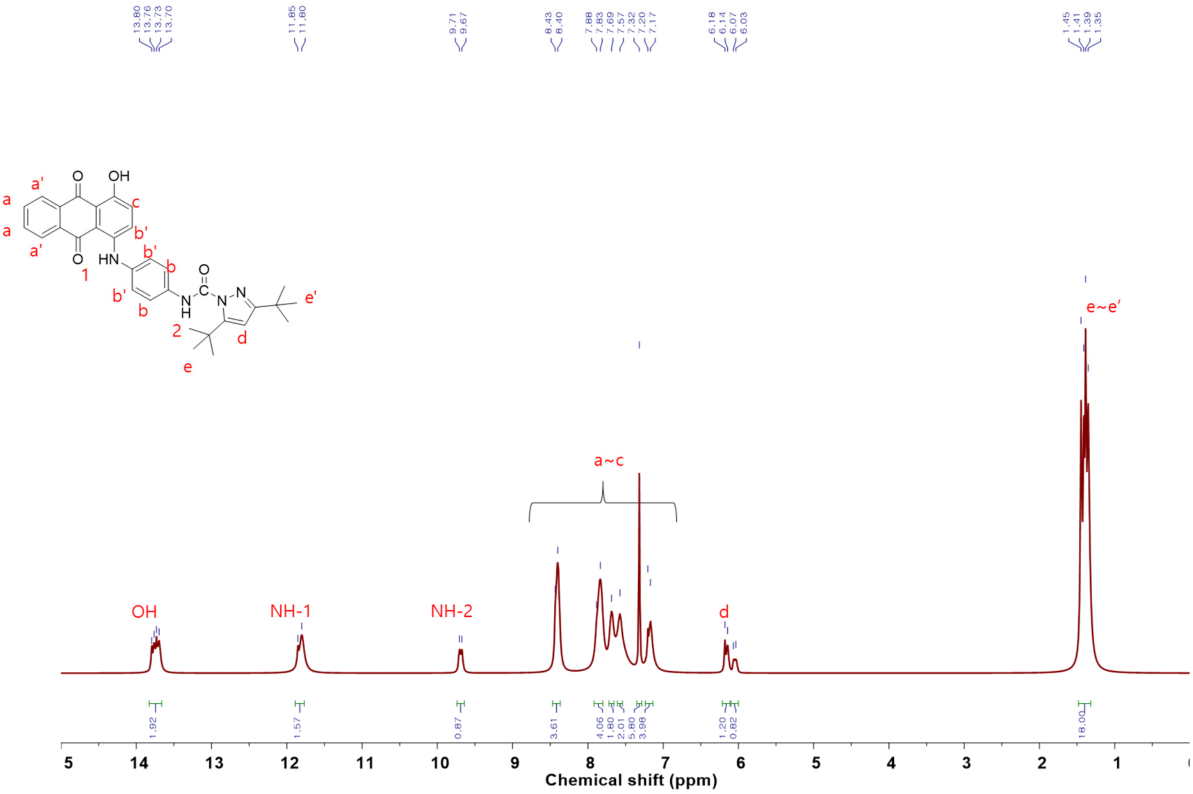

Figure S5. H-NMR spectrum of DTP-A
